# Supplementary material for: Improving Plasmonic Photothermal Therapy of Lung Cancer Cells with Anti-EGFR Targeted Gold Nanorods
Source: Nanomaterials (Basel). 2020 Jul 3;10(7):1307. doi: 10.3390/nano10071307 (PMC7407807; doi:10.3390/nano10071307)
Supplement: Supplementary file 1 [file nanomaterials-10-01307-s001.pdf]

Supplementary Materials: Improving plasmonic photothermal therapy of lung cancer cells with anti-EGFR targeted gold nanorods

Oscar Knights [1], Steven Freear [1] and James R. McLaughlan [1,2,*]

1. Laser type: continuous wave vs pulsed for plasmonic photothermal therapy

There are broadly two categories of lasers used for photothermal therapy: pulsed (PW) and continuous wave (CW). CW lasers emit a continuous beam of light that generally lasts seconds or longer. The laser medium is continuously pumped by another light-source (usually another laser) resulting in the continual excitation of atoms within the lasing medium and the emission of photons [1]. CW lasers are also relatively inexpensive compared with most pulsed laser systems, while also having the advantage of compactness and portability.

High power pulsed lasers (PW), in contrast, are usually complex, bulky systems with multiple components such as gas tanks, movable crystals, and liquid cooling units, requiring longer, more sophisticated calibration and installation times. Unlike CW lasers that have a steady, constant power output, PW lasers frequently have intra-cavity delays that are built into the laser system to enable the build-up and 'storage' of energy in the lasing medium until it is released as a high-intensity burst of light [2]. Since there is a delay between exciting the lasing medium and a light pulse being emitted from the shutter, high power PW lasers can usually only achieve pulse repetition frequencies (PRFs) of around $10-20$ Hz. The process of rapid accumulation and release of energy in very short, nano- or femto-second pulses is termed "Q-switching" and facilitates the lasers ability to generate extremely high peak powers in the order of 10^6 to 10^9 Watts. This is because the peak power, P_{peak}, is the energy transfer of a single laser pulse, given by,

$$P_{\text{peak}}(W) = \frac{E_{\text{pulse}}}{\tau_p} \tag{1}$$

where E_{pulse} is the energy of a single pulse, and τ_p is the duration of the pulse. The average power, P_{ave}, of a pulsed laser takes into account the time between each pulse by the following equation,

$$P_{\text{ave}}(W) = \frac{E_{\text{pulse}}}{\tau_T} = E_{\text{pulse}} \times \text{PRF(Hz)} \tag{2}$$

where $\tau_T = 1/\text{PRF}$ is the time-period of the pulses. This means for a single, 7 ns pulse with an energy of 1 J and PRF $=$ 10 Hz, the peak power is 143×10^6 W and the average power is only 10 W. Using equation 2 gives an $P_{\text{ave}} = 160$ mW for the PW laser and 950 mW for the CW laser.

The fundamental differences between CW and PW lasers lead to significant differences in the way they interact with materials. When a laser interacts with a material, and is absorbed, the energy first excites the conduction electrons and sets them oscillating [3]. This energy is then transferred to the surrounding electrons via femtosecond electron-electron relaxations, before being further transferred to the lattice, via electron-phonon collisions on a picosecond time-scale. When a material absorbs light from a CW laser, the heat is spread out over a much larger volume since the continuous nature of the incoming light allows for the heat to diffuse radially [4]. If the absorbing material is a solution of AuNRs, then this effect will be significantly enhanced as the AuNRs efficiently convert the laser energy into heat [5]. Conversely, under pulsed laser illumination, the short ($<$ 10 ns) high intensity laser pulses induce a higher initial peak temperature in the absorbers, compared with that of CW lasers. However, due to the time period between laser pulses, the absorbing region has time to cool sufficiently before each consecutive pulse. If AuNRs are the absorbing medium, then they will experience extremely high peak temperatures (and the emission of PA signals if the stress confinement condition is met), before rapidly cooling ahead of the next pulse (supplementary figure S1). Thus, two distinct mechanisms

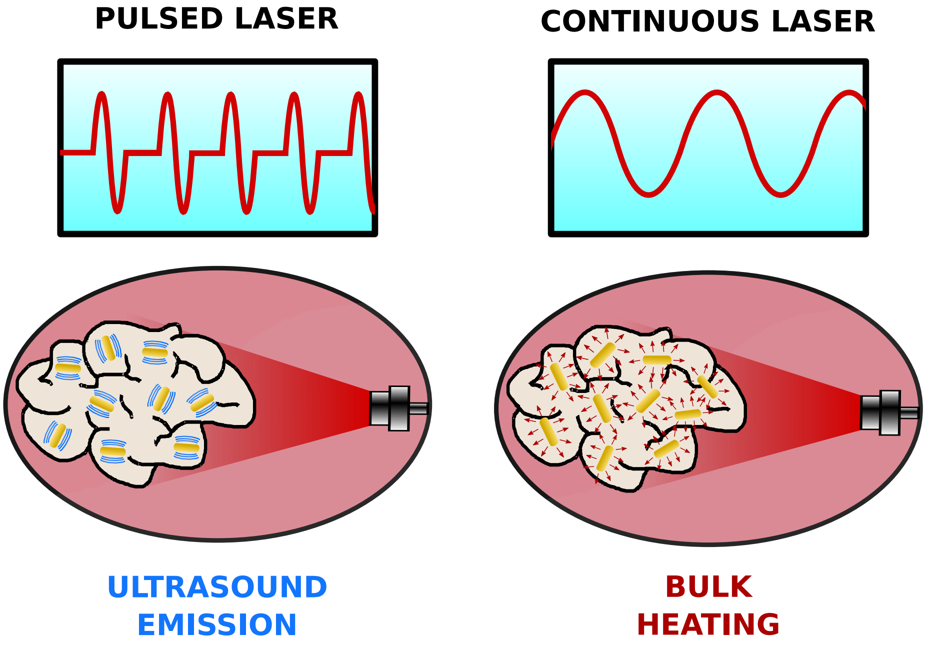

Figure S1. Under pulsed laser illumination, the AuNRs located in a tumour will experience a rapid increase in temperature that likely causes an expansion of both the AuNR and the localised surroundings, resulting in the emission of an ultrasonic wave. Conversely, under continuous wave laser illumination, the same AuNRs will continuously radiate heat to the surroundings, causing bulk heating of the environment.

exist depending on the type of laser employed: highly localised heating when PW lasers are used, and bulk, volumetric heating when CW lasers are used. There are also differences in the guidelines for the maximum exposure limit for skin. The maximum permissible exposure (MPE) is governed by the optical wavelength, λ, and duration of the laser exposure, $t(\mathrm{s})$ [6]. Within the wavelength range $700\,\mathrm{nm} \leq \lambda \leq 1400\,\mathrm{nm}$, the MPE for skin is determined by the following,

$$\mathrm{MPE_{skin}} = \begin{cases} 2 \times 10^{11}\, C_4 \,(\mathrm{W\,m^{-2}}), & \text{if } t(s) < 10^{-9} \\ 200\, C_4 \,(\mathrm{J\,m^{-2}}), & \text{if } 10^{-9} \leq t(s) \leq 10^{-7} \\ 1.1 \times 10^{4}\, C_4\, t(s)^{0.25} \,(\mathrm{J\,m^{-2}}), & \text{if } 10^{-7} \leq t(s) \leq 10 \\ 2000\, C_4 \,(\mathrm{W\,m^{-2}}), & \text{if } 10 \leq t(s) \leq 30000 \end{cases} \tag{3}$$

where C_4 is a wavelength-dependent constant given by,

$$C_4 = \begin{cases} 10^{0.002(\lambda-700)}, & \text{for } 700\,\mathrm{nm} \leq \lambda \leq 1050\,\mathrm{nm} \\ 5, & \text{for } 1050\,\mathrm{nm} \leq \lambda \leq 1400\,\mathrm{nm} \end{cases} \tag{4}$$

This relationship between laser wavelength and exposure duration gives rise to a different MPE depending on the laser employed. As the length of laser exposure is increased from a nanosecond pulse (10^{-9}) to a duration of 10 s, the MPE for skin increases from approximately 400 to 38 000 $\mathrm{J\,m^{-2}}$ (using equations 3 and 4 with a laser wavelength = 850 nm). This suggests that short pulses of light are more damaging to skin than a prolonged energy deposition. For exposures longer than 10 s, the defining limit changes from the total energy per unit area delivered to the skin ($\mathrm{J\,m^{-2}}$) to the total power per unit area ($\mathrm{W\,m^{-2}}$), and becomes a function of wavelength only. As the wavelength is increased from 700 to 1400 nm, the MPE for skin increases from approximately 2010 to 10 000 $\mathrm{W\,m^{-2}}$, for exposures lasting more than 10 s, indicating that the longer NIR optical wavelengths are less

damaging to skin. Since CW lasers are predominately used on the order of minutes (for example in photothermal therapy), their MPE limits are measured in terms of the total power per unit area, with a limit of $MPE_{skin} = 4000\,W\,m^{-2}$ ($\lambda = 850$). However, a PW laser employed for photoacoustics will operate with a pulse length on the order of a few nanoseconds and therefore its MPE is measured in terms of the total energy per unit area. For a 7 ns pulse at 850, its $MPE_{skin} = 400\,J\,m^{-2}$. While these guidelines provide a potential upper limit on the safe exposure for skin, they do not cover other biological tissues (other than ocular exposure) that may be exposed during therapy, such as lung tissue, and therefore they should be taken as a reference guideline only for future therapeutic development.

PPTT is conventionally administered with CW lasers to induce hyperthermia via bulk heating of a target region. This volumetric elevation in temperature can lead to the death of cells via two basic pathways: apoptosis and necrosis. Necrosis is the death of cells related to injury, disease, or low blood supply, and typically results in the loss of membrane integrity and the uncontrolled release of intracellular material into the surroundings [7]. Apoptosis differs from necrosis in that it is a form of controlled cell death, where a series of specific cellular biochemical and morphological events occur that ultimately lead to the 'programmed' elimination of ageing, superfluous, or damaged cells [8]. This method for cell death is a natural way for the body to eliminate unwanted or unneeded cells that are no longer operating as normal. If cells experience an apoptotic death, then no immunogenic or inflammatory response will be observed [9]. Conversely, if cells are destroyed via a necrotic pathway, then the release of cellular contents will initiate an inflammatory response. The cellular mechanisms and processes that govern necrosis and apoptosis have been discussed in great detail in the literature and so will not be discussed here [10,11].

With regards to laser-based therapies, the conditions that influence whether cells undergo either necrosis or apoptosis vary based on the laser exposure parameters and whether absorbing agents, such as AuNRs, are used. It has been shown that the threshold for apoptosis and necrosis of human prostate cancer cells is between 44 °C or 45 °C when maintained at this temperature for 120 min [12]. However, this study was not performed using a laser as the heating source and instead was conducted by placing a 96-well plate onto a pre-heated hotplate for heating. This method for applying heat may result in a different temperature profile compared with that of a laser, and so comparisons must be made with caution. Another more recent study investigated CW laser-induced cell-death of a murine melanoma cell line that was incubated with AuNRs and exposed to 15 min laser irradiation [13]. It was found that, when heated to a maximum of 43 °C after 15 min laser exposure, the majority of the cells survived. However, when temperatures between $43 - 49$ °C were reached, significant cell death was observed (approximately 80 %) and the primary mechanism was apoptosis. Above 49 °C, necrosis became the leading cause of cell death. This study was consistent with previous reports on temperature thresholds, where temperatures above 50 °C were shown to induce necrosis, and temperatures below this threshold primarily induced apoptosis [14,15]. The strong temperature dependence on the mechanisms for cell-death give rise to the ability for CW lasers to selectively destroy tissues via either the necrotic or apoptotic pathways, by simply controlling laser exposure parameters.

Unlike CW lasers, PW lasers do not induce bulk temperature changes in an absorbing region, and therefore the mechanisms for destroying tissue are different. The absorption of high-intensity light pulses likely result in the destruction of tissue via mechanical stresses and bubble cavitation [16,17]. As a result of this, pulsed-wave plasmonic photothermal therapy (PW-PPTT) (a.k.a. photoacoustic therapy) can only induce necrotic cell-death, since the mechanical stresses destroy the cells likely by disrupting the cell membrane and releasing the intracellular contents into the surroundings.

Although apoptosis is generally preferred over necrosis, due to the lack of immunogenic or inflammatory response, cell-death via necrosis has its potential benefits, such as the immediacy of the killing effect, no risks associated with cancer cells developing resistance to the therapy [18], increased selectivity as a result of no heat conducting from the target site and damaging surrounding healthy tissues, potentially lower laser powers compared with CW lasers, and the ability for the generated PA signals to provide simultaneous imaging during treatment [19]. Furthermore, PW-PPTT may enable

the ability to combine PAI with PPTT through the use of PW lasers, and incorporate a single laser system into already existing medical technologies [20], ultimately improving patient outcome and treatment efficiency.

1. Paschotta, R. *Field Guide to Lasers*; SPIE Press Book, 2008.
2. Siegman, A. *Lasers*; University Science Books, 1986.
3. Han, J.; Li, Y. *Lasers - Applications in Science and Industry*; InTech, 2011.
4. Harris-Birtill, D.; Singh, M.; Zhou, Y.; Shah, A.; Ruenraroengsak, P.; Gallina, M.E.; Hanna, G.B.; Cass, A.E.; Porter, A.E.; Bamber, J.; others. Gold nanorod reshaping in vitro and in vivo using a continuous wave laser. *PloS one* **2017**, *12*, e0185990.
5. Jain, S.; Hirst, D.; O'sullivan, J. Gold nanoparticles as novel agents for cancer therapy. *The British journal of radiology* **2012**, *85*, 101–113.
6. American National Standard for Safe Use of Lasers. ANSI. *ANSI* **2014**, *Z136.1*.
7. Niemann, B.; Rohrbach, S. Metabolically Relevant Cell Biology–Role of Intracellular Organelles for Cardiac Metabolism. In *The Scientist's Guide to Cardiac Metabolism*; Elsevier, 2016; pp. 19–38.
8. Tanase, C.P.; Ogrezeanu, I.; Badiu, C. 5 - Apoptosis. In *Molecular Pathology of Pituitary Adenomas*; Tanase, C.P.; Ogrezeanu, I.; Badiu, C., Eds.; Elsevier: London, 2012; pp. 45 – 52.
9. Pérez-Hernández, M.; del Pino, P.; Mitchell, S.G.; Moros, M.; Stepien, G.; Pelaz, B.; Parak, W.J.; Gálvez, E.M.; Pardo, J.; de la Fuente, J.M. Dissecting the molecular mechanism of apoptosis during photothermal therapy using gold nanoprisms. *ACS nano* **2014**, *9*, 52–61.
10. Kroemer, G.; Dallaporta, B.; Resche-Rigon, M. The mitochondrial death/life regulator in apoptosis and necrosis. *Annual review of physiology* **1998**, *60*, 619–642.
11. Kiraz, Y.; Adan, A.; Yandim, M.K.; Baran, Y. Major apoptotic mechanisms and genes involved in apoptosis. *Tumor Biology* **2016**, *37*, 8471–8486.
12. Song, A.S.; Najjar, A.M.; Diller, K.R. Thermally induced apoptosis, necrosis, and heat shock protein expression in three-dimensional culture. *Journal of biomechanical engineering* **2014**, *136*, 071006.
13. Zhang, Y.; Zhan, X.; Xiong, J.; Peng, S.; Huang, W.; Joshi, R.; Cai, Y.; Liu, Y.; Li, R.; Yuan, K.; others. Temperature-dependent cell death patterns induced by functionalized gold nanoparticle photothermal therapy in melanoma cells. *Scientific reports* **2018**, *8*, 8720.
14. Huang, X.; Jain, P.K.; El-Sayed, I.H.; El-Sayed, M.A. Determination of the minimum temperature required for selective photothermal destruction of cancer cells with the use of immunotargeted gold nanoparticles. *Photochemistry and photobiology* **2006**, *82*, 412–417.
15. Ali, M.R.; Rahman, M.A.; Wu, Y.; Han, T.; Peng, X.; Mackey, M.A.; Wang, D.; Shin, H.J.; Chen, Z.G.; Xiao, H.; others. Efficacy, long-term toxicity, and mechanistic studies of gold nanorods photothermal therapy of cancer in xenograft mice. *Proceedings of the National Academy of Sciences* **2017**, *114*, E3110–E3118.
16. Pitsillides, C.M.; Joe, E.K.; Wei, X.; Anderson, R.R.; Lin, C.P. Selective Cell Targeting with Light-Absorbing Microparticles and Nanoparticles. *Biophysical Journal* **2003**, *84*, 4023 – 4032. doi:http://dx.doi.org/10.1016/S0006-3495(03)75128-5.
17. Tong, L.; Zhao, Y.; Huff, T.B.; Hansen, M.N.; Wei, A.; Cheng, J.X. Gold nanorods mediate tumor cell death by compromising membrane integrity. *Advanced Materials* **2007**, *19*, 3136–3141.
18. Melamed, J.R.; Edelstein, R.S.; Day, E.S. Elucidating the fundamental mechanisms of cell death triggered by photothermal therapy. *ACS nano* **2015**, *9*, 6–11.
19. Zhong, J.; Wen, L.; Yang, S.; Xiang, L.; Chen, Q.; Xing, D. Imaging-guided high-efficient photoacoustic tumor therapy with targeting gold nanorods. *Nanomedicine: Nanotechnology, Biology and Medicine* **2015**, *11*, 1499–1509.
20. Knights, O.B.; Ye, S.; Ingram, N.; Freear, S.; McLaughlan, J.R. Optimising gold nanorods for photoacoustic imaging in vitro. *Nanoscale Advances* **2019**.
